# Changes in blood Krebs von den Lungen-6 predict the mortality of patients with acute exacerbation of interstitial lung disease

**DOI:** 10.1038/s41598-022-08965-9

**Published:** 2022-03-22

**Authors:** Myeong Geun Choi, Sun Mi Choi, Jae Ha Lee, Jung-Ki Yoon, Jin Woo Song

**Affiliations:** 1 Department of Pulmonary and Critical Care Medicine, Asan Medical Center, University of Ulsan College of Medicine, 88, Olympic-ro 43-gil, Songpa-gu, Seoul, 05505 South Korea; 2 Division of Pulmonary and Critical Care Medicine, Department of Internal Medicine, Seoul National University Hospital, Seoul National University College of Medicine, Seoul, South Korea; 3 Division of Pulmonary and Critical Care Medicine, Department of Internal Medicine, Inje University Haeundae Paik Hospital, Inje University College of Medicine, Busan, South Korea

**Keywords:** Disease-free survival, Prognostic markers

## Abstract

Acute exacerbation (AE) significantly affects the prognosis of patients with interstitial lung disease (ILD). This study aimed to investigate the best prognostic biomarker for patients with AE-ILD. Clinical data obtained during hospitalization were retrospectively analyzed for 96 patients with AE-ILD at three tertiary hospitals. The mean age of all subjects was 70.1 years; the percentage of males was 66.7%. Idiopathic pulmonary fibrosis accounted for 60.4% of the cases. During follow-up (median: 88 days), in-hospital mortality was 24%. Non-survivors had higher lactate dehydrogenase and C-reactive protein (CRP) levels, lower ratio of partial pressure of oxygen to the fraction of inspiratory oxygen (P/F ratio), and higher relative change in Krebs von den Lungen-6 (KL-6) levels over 1 week after hospitalization than survivors. In multivariable analysis adjusted by age, the 1-week change in KL-6—along with baseline P/F ratio and CRP levels—was an independent prognostic factor for in-hospital mortality (odds ratio 1.094, *P* = 0.025). Patients with remarkable increase in KL-6 (≥ 10%) showed significantly worse survival (in-hospital mortality: 63.2 vs. 6.1%) than those without. In addition to baseline CRP and P/F ratio, the relative changes in KL-6 over 1 week after hospitalization might be useful for predicting in-hospital mortality in patients with AE-ILD.

## Introduction

Interstitial lung disease (ILD) is a heterogeneous group of disorders characterized by inflammation and/or fibrosis involving pulmonary interstitium^1,2^. The clinical course of ILD is highly variable, and acute exacerbation (AE) is a fatal complication with high mortality^3^. The annual incidence of AE-ILD varies in the range of 4–15% according to the type of ILD or study population^4–6^. Based on previous studies, in-hospital mortality was up to 50% and no significant difference in in-hospital mortality was found between idiopathic pulmonary fibrosis (IPF) and non-IPF ILD^4,5,7^. Previous studies revealed several predicting factors for mortality in patients with AE-ILD; low baseline forced vital capacity (FVC) and diffusion of carbon monoxide (DLco) before AE, extensive ground-glass opacity or consolidation on high-resolution computed tomography (HRCT), impaired oxygenation, high lactate dehydrogenase (LDH) and C-reactive protein (CRP) levels, and low percentages of lymphocytes on bronchoalveolar lavage (BAL) fluid at the time of hospitalization were associated with poor prognosis of patients with AE-ILD^4,8–10^.

In contrast to the pulmonary function test, HRCT, or BAL fluid analysis, blood biomarkers are relatively easy to test independent of patient effort or reader ability and can be measured less invasively^11^. Krebs von den Lungen-6 (KL-6) is a mucin-like glycoprotein produced by type 2 alveolar epithelial cells that is released into the bloodstream through the damaged alveolar basement membrane when type 2 alveolar epithelial cells are injured and proliferated^12,13^. Recently, some studies have revealed that baseline KL-6 levels at hospitalization are associated with mortality in AE-ILD^8,14^. KL-6 was also reported to be useful for predicting disease severity, clinical course, and prognosis of patients with ILD^12,15,16^. However, no studies have compared changes in blood biomarkers, including KL-6, in patients with AE-ILD and investigated their role in predicting the prognosis of patients with AE-ILD. Therefore, the aim of this study was to identify the best marker by comparing the usefulness of blood biomarkers for predicting the prognosis of patients with AE-ILD.

## Methods

### Study population

**Figure 1:**
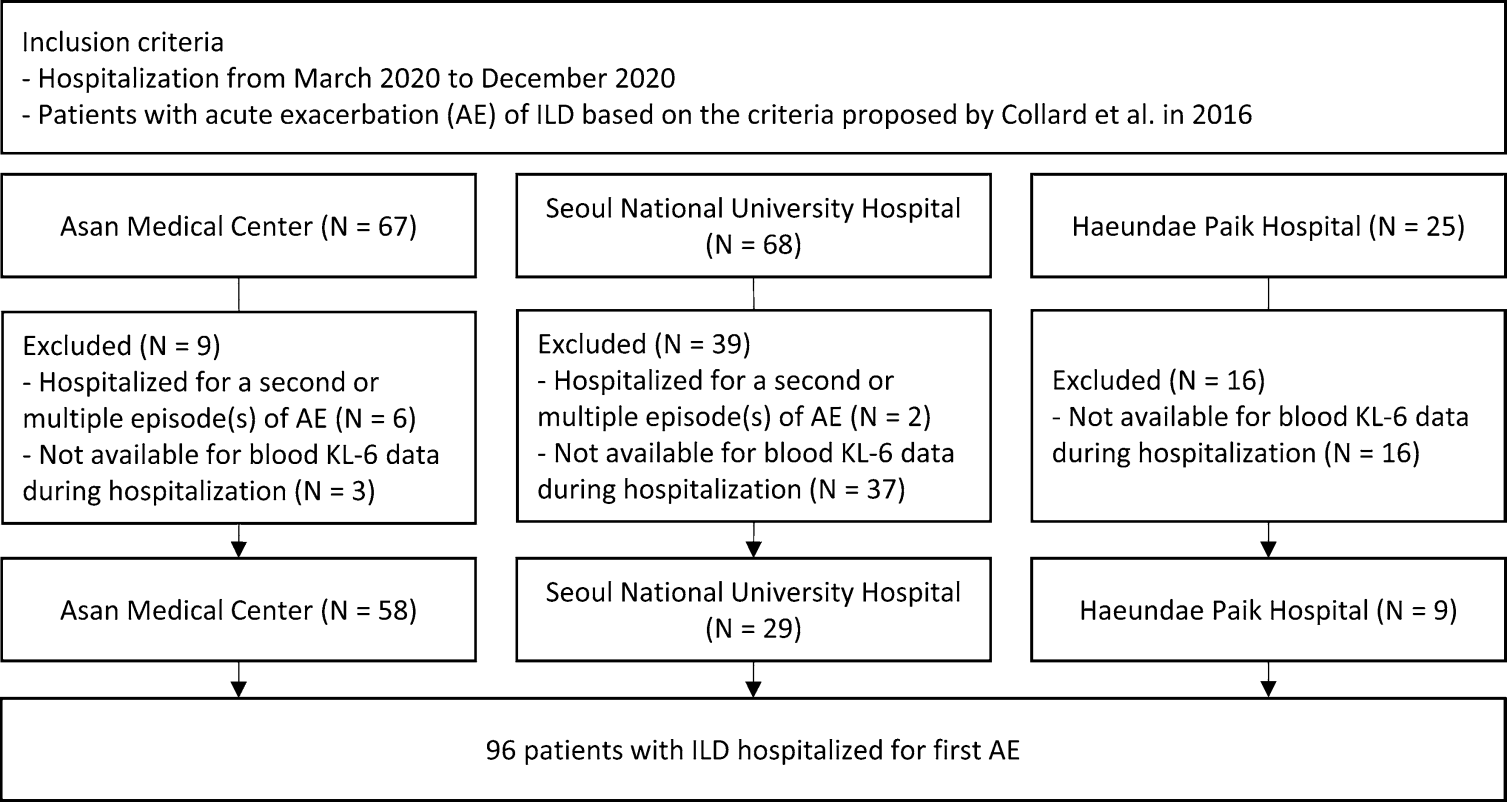
Flowchart of patient selection. *ILD* interstitial lung disease, *AE* acute exacerbation, *KL-6* Krebs von den Lungen-6.

From March 2020 to December 2020, 160 patients with ILD, who were hospitalized due to AE at three tertiary hospitals in South Korea, were screened for enrolment in this study. Of the 160 patients, 8 patients who were hospitalized because of a second or multiple episode (s) of AE and 56 patients without blood KL-6 data during hospitalization were excluded. Finally, 96 patients with AE-ILD (58 at Asan Medical Center, 29 at Seoul National University Hospital, and 9 at Haeundae Paik Hospital) were included in this study (Fig. 1). ILD was diagnosed according to international guidelines^1,2,17,18^.

This study was approved by the Institutional Review Board of the Asan Medical Center (IRB No. 2021-0263), the Seoul National University Hospital (IRB No. H-2107-046-1233), and Haeundae Paik Hospital (IRB No. 2021-05-018) and conducted in accordance with the ethical standards of the Declaration of Helsinki. The requirement for written informed consent was waived owing to the retrospective nature of this study. All methods were performed in accordance with the relevant guidelines and regulations.

### Data collection

Clinical and survival data for all patients during hospitalization were obtained from medical records and/or the National Health Insurance of Korea records. The results of pulmonary function test (PFT) and 6-min walk test (6MWT) within 3 months of hospitalization were also collected and considered baseline values. Spirometry was performed, and DL_CO_ and total lung capacity (TLC) were measured according to the American Thoracic Society/European Respiratory Society recommendations^19–21^. The results are expressed as percentages of the normal predicted values. The BAL fluid analysis and 6MWT were performed in accordance with previously published guidelines^22,23^. Based on the criteria suggested by Collard et al., AE was defined as acute worsening of dyspnea typically within 30 days, with new bilateral lung infiltration that is not fully explained by heart failure or fluid overload and without any identified extra-parenchymal causes (pneumothorax, pleural effusion, or pulmonary embolism)^5^.

The Nanopia KL-6 assay (SEKISUI MEDICAL, Tokyo, Japan) was used to measure blood KL-6 levels. All samples were immediately transported and centrifuged after blood collection. The latex-enhanced immunoturbidimetric assay, which measures changes in absorbance by agglutination, was used to measure KL-6 concentration. CRP and LDH levels were measured using Cobas 8000 (ROCHE DIAGNOSTICS, Basel, Switzerland). The partial pressure of oxygen to the fraction of inspiratory oxygen ratio (P/F ratio) was calculated using the arterial blood gas analysis results obtained with pHOX Ultra (NOVA BIOMEDICAL, Waltham, MA, USA).

### Statistical analysis

Continuous variables are expressed as mean ± standard deviation and were compared using an unpaired Student’s *t*-test. Categorical variables are presented as frequency and percentage and were compared using Chi-squared and Fisher’s exact tests. Absolute and relative changes in blood markers over 1 week from the baseline values were calculated as follows: absolute changes = measurement at follow-up − measurement at baseline; and relative changes = (measurement at follow-up − measurement at baseline)/measurement at baseline × 100 (%). Survival was analyzed using the Kaplan–Meier method, and the difference was assessed via a log-rank test. A logistic regression analysis was used to identify risk factors for in-hospital mortality in patients with AE. Variables with a *P* value of less than 0.1 in the unadjusted analysis were selected and adjusted by age for the multivariable analysis. Using the receiver operating characteristic (ROC) curve analysis, the best cut-off level of variables that predict in-hospital mortality was determined. A *P* value of less than 0.05 was considered significant. Statistical analyses were performed using IBM SPSS version 25.0 (IBM Corp., Armonk, NY, USA).

## Results

### Baseline characteristics

The mean age of the total patients was 70.1 years, the percentage of males was 66.7%, and 60.4% of patients had IPF (Table 1). Triggered and idiopathic AE were 32.3% and 67.7%, respectively. The baseline characteristics of patients were similar among the three hospitals, except for body mass index (Supplementary Table S1). During the follow-up period (median 88 days, interquartile range 24–176 days), 42 (43.8%) patients died after hospitalization; in-hospital mortality rate was 24.0% (n = 23). The 30-day and 90-day mortality rates were 21.6% and 36.1%, respectively. There was no difference in survival after hospitalization (the median survival: 134 days vs. not reached, P = 0.250) between patients with AE-IPF and those with AE-non IPF (Supplementary Figure S1). At the time of hospitalization, the non-survivors showed higher LDH and CRP levels and lower P/F ratio than the survivors (Table 1); however, there was no difference in baseline KL-6 levels and other clinical variables between the two groups. During hospitalization, almost all patients (99.0%) received steroid treatment (the median dose: 55.9 mg), and steroid pulse therapy and cytotoxic agents were provided in 45.8% and 12.5%, respectively (Table 2).

**Table 1:** Comparison of the baseline characteristics between non-survivors and survivors during hospitalization.

	Total	Non-survivors	Survivors	*P* value
Number of patients	96	23	73	
Age, year	70.1 ± 9.4	73.1 ± 8.6	69.1 ± 9.4	0.076
Male	64 (66.7)	16 (69.6)	48 (65.8)	0.804
Ever-smoker	60 (62.5)	13 (56.5)	47 (64.4)	0.801
BMI, kg/m^2^	23.4 ± 3.6	22.4 ± 3.2	23.7 ± 3.6	0.144
**Diagnosis**				0.278
IPF	58 (60.4)	15 (65.2)	43 (58.9)	
CTD-ILD	17 (17.7)	5 (21.7)	12 (16.4)	
HP	4 (4.2)	0 (0.0)	4 (5.5)	
Unclassifiable	17 (17.7)	3 (13.0)	14 (19.2)	
**Pulmonary function test**^**a**^** (n = 57)**		(n = 11)	(n = 46)	
FVC, % predicted	53.9 ± 16.5	55.5 ± 16.3	53.5 ± 16.7	0.731
TLC, % predicted	58.6 ± 14.1	58.4 ± 13.6	58.7 ± 14.4	0.959
DLco, % predicted	37.4 ± 16.9	30.8 ± 8.9	39.3 ± 18.2	0.143
**6-min walk test**^**a**^** (n = 47)**		(n = 9)	(n = 38)	
Distance, m	295.6 ± 143.0	326.2 ± 164.2	288.3 ± 138.9	0.480
Lowest SpO2, %	84.1 ± 6.2	83.3 ± 4.9	84.3 ± 6.5	0.675
**BAL fluid analysis (n = 38)**		(n = 7)	(n = 31)	
BAL, neutrophil, %	34.7 ± 28.3	44.7 ± 24.8	32.5 ± 28.9	0.308
BAL, lymphocyte, %	12.9 ± 15.0	7.9 ± 5.3	14.1 ± 16.3	0.330
**Blood biomarkers**				
KL-6, U/mL	1732.4 ± 1112.7	1754.4 ± 1475.0	1726.0 ± 995.6	0.919
LDH, IU/L	402.6 ± 165.8	479.7 ± 192.3	373.6 ± 146.7	0.019
CRP, mg/dL	6.1 ± 7.7	12.1 ± 8.9	4.2 ± 6.1	< 0.001
P/F ratio	289.9 ± 127.4	224.5 ± 162.7	312.8 ± 104.9	0.032
Use of antifibrotic agents^a^	60 (62.5)	13 (56.5)	47 (64.4)	0.497

Data are presented as mean ± standard deviation or number (%).

*BMI* body mass index, *IPF* idiopathic pulmonary fibrosis, *CTD* connective tissue disease, *ILD* interstitial lung disease, *HP* hypersensitivity pneumonitis, *FVC* forced vital capacity, *TLC* total lung capacity, *DLco* diffusing capacity of the lung for carbon monoxide, *BAL* bronchoalveolar lavage, *KL-6* Krebs von den Lungen-6, *LDH* lactate dehydrogenase, *CRP* C-reactive protein, *P/F ratio* ratio of partial pressure of oxygen to the fraction of inspiratory oxygen.

^a^At the time of hospitalization.

**Table 2:** Comparison of treatment for AE-ILD during hospitalization.

	Total	Non-survivors	Survivors	*P* value
Number of patients	96	23	73	
Steroid	95 (99.0)	23 (100)	72 (98.6)	0.573
Initial dose of steroid^a^	55.9 (36.8–74.9)	60.0 (40–500)	55.1 (35.8–67.2)	0.186
Steroid pulse^b^	44 (45.8)	11 (47.8)	33 (45.2)	0.826
Cytotoxic agent^c^	12 (12.5)	3 (13.0)	9 (12.3)	0.928

Data are presented as median (interquartile range) or number (%).

*AE-ILD* acute exacerbation of interstitial lung disease.

^a^Methylprednisolone (mg); ^b^≥ 500 mg/day methylprednisolone for 3 days; ^c^Azathioprine, cyclosporine, or cyclophosphamide.

### Changes in blood biomarkers

The absolute and relative changes in KL-6 levels over 1 week after hospitalization were significantly higher in non-survivors than in survivors (Table 3). However, there were no differences in the changes in LDH, CRP, and P/F ratio levels over 1 week between the two groups.

**Table 3:** Comparison of changes in the blood biomarkers from baseline to 1 week between non-survivors and survivors during hospitalization.

	Non-survivors	Survivors	*P* value
**Changes from baseline (1-week)**			
KL-6, absolute, U/mL	+ 565.2 ± 683.5	− 22.1 ± 258.2	0.007
KL-6, relative, %	+ 67.5 ± 77.5	+ 1.5 ± 12.5	0.007
LDH, absolute, IU/L	+ 36.6 ± 142.4	− 77.6 ± 80.2	0.063
LDH, relative, %	+ 19.5 ± 39.6	− 14.9 ± 17.9	0.095
CRP, absolute, mg/dL	− 6.8 ± 8.2	− 2.8 ± 3.4	0.057
CRP, relative, %	− 33.2 ± 101.6	− 55.5 ± 81.0	0.350
P/F ratio, absolute	− 51.8 ± 190.5	+ 7.1 ± 96.4	0.242
P/F ratio, relative, %	+ 54.3 ± 154.1	+ 6.5 ± 29.7	0.223

Data are presented as mean ± standard deviation.

*KL-6* Krebs von den Lungen-6, *LDH* lactate dehydrogenase, *CRP* C-reactive protein, *P/F ratio* ratio of partial pressure of oxygen to the fraction of inspiratory oxygen.

### Risk factors for in-hospital mortality

In the unadjusted logistic regression analysis, CRP, P/F ratio, and relative changes in KL-6 levels over 1 week were identified as significant risk factors for the in-hospital mortality of patients with AE-ILD (Table 4). In the multivariable analysis adjusted by age, CRP, P/F ratio, and changes in KL-6 levels were also independent prognostic factors for in-hospital mortality. When classified according to the type of ILD, baseline LDH and relative changes in KL-6 levels over 1 week were significantly associated with in-hospital mortality in patients with AE-IPF in the unadjusted logistic regression analysis (Supplementary Table S2); however, baseline CRP and changes in CRP levels over 1 were significant prognostic factors in those with AE-non IPF (Supplementary Table S3).

**Table 4:** Logistic regression analysis for in-hospital mortality in patients with AE-ILD.

Variables	Unadjusted analysis			Multivariable analysis adjusted by age		
	Odds ratio	95% CI	*P* value	Odds ratio	95% CI	*P* value
Age	1.051	0.994–1.112	0.080		–	
Male	1.190	0.433–3.273	0.735		–	
Ever-smoker	0.799	0.301–2.120	0.652		–	
IPF (vs. non-IPF)	1.308	0.493–3.474	0.590		–	
FVC, % predicted	1.007	0.968–1.048	0.725		–	
TLC, % predicted	0.998	0.944–1.056	0.958		–	
DLco, % predicted	0.962	0.914–1.013	0.146		–	
6MWT, distance, m	1.002	0.997–1.007	0.472		–	
6MWT, SpO2, %	0.974	0.864–1.098	0.667		–	
BAL, neutrophil, %	1.015	0.986–1.045	0.303		–	
BAL, lymphocyte, %	0.953	0.862–1.054	0.346		–	
Baseline KL-6	1.000	1.000–1.000	0.918		–	
Baseline LDH	1.004	1.000–1.007	0.026	1.004	1.000–1.008	0.056
Baseline CRP	1.146	1.062–1.236	< 0.001	1.214	1.056–1.394	0.006
Baseline P/F ratio	0.994	0.989–0.999	0.010	0.990	0.982–0.997	0.005
Δ KL-6, 1 week	1.007	1.002–1.013	0.007	1.007	1.000–1.015	0.066
Δ KL-6, 1 week, %	1.109	1.039–1.184	0.002	1.094	1.011–1.183	0.025
Δ LDH, 1 week	1.011	1.000–1.021	0.045	1.015	0.998–1.031	0.079
Δ LDH, 1 week, %	1.044	1.004–1.086	0.031	1.050	0.997–1.105	0.064
Δ CRP, 1 week	0.873	0.786–0.970	0.012	1.039	0.867–1.245	0.680
Δ CRP, 1 week, %	1.003	0.997–1.008	0.368		–	
Δ P/F ratio, 1 week	0.997	0.992–1.001	0.144		–	
Δ P/F ratio, 1 week, %	1.006	0.999–1.013	0.112		–	
Use of antifibrotic agents^a^	0.719	0.277–1.866	0.498		–	
Steroid pulse^b^	1.111	0.434–2.842	0.826		–	
Cytotoxic agent^b^	1.067	0.263–4.325	0.928		–	

^a^At the time of hospitalization; ^b^treatment for AE during hospitalization.

*AE-ILD* acute exacerbation of interstitial lung disease, *IPF* idiopathic pulmonary fibrosis, *FVC* forced vital capacity, *TLC* total lung capacity, *DLco* diffusing capacity of the lung for carbon monoxide, *6MWT* 6-min walk test, *BAL* bronchoalveolar lavage, *KL-6* Krebs von den Lungen-6, *LDH* lactate dehydrogenase, *CRP* C-reactive protein, *P/F ratio* ratio of partial pressure of oxygen to the fraction of inspiratory oxygen, *Δ* changes from baseline.

**Figure 2:**
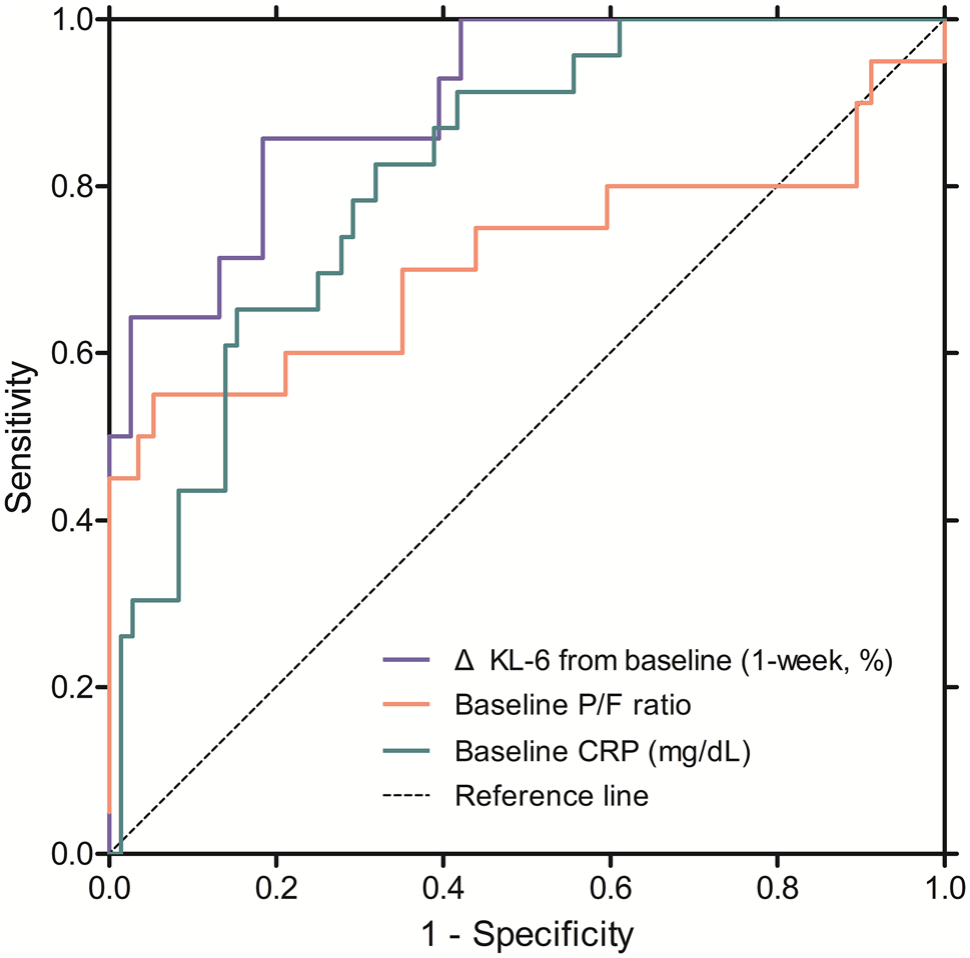
Comparison of the receiver operating characteristic curve of blood markers for predicting in-hospital mortality in patients with AE-ILD. *AE-ILD* acute exacerbation of interstitial lung disease; ROC curves: blue line, KL-6 relative change from baseline; red line, baseline P/F ratio; green line, baseline CRP. *KL-6* Krebs von den Lungen-6, *P/F ratio* ratio of partial pressure of oxygen to the fraction of inspiratory oxygen, *CRP* C-reactive protein, *AUC* area under the curve.

In the ROC curve analysis, CRP, P/F ratio, and the relative changes in KL-6 levels over 1 week were useful for predicting in-hospital mortality; however, changes in KL-6 levels showed the highest area under the curve (AUC) values (0.902 [*P* < 0.001] vs. 0.818 [CRP, *P* < 0.001], 0.713 [P/F ratio, *P* = 0.005]) (Fig. 2 and Table 5). The best cut-off level for changes in KL-6 over 1 week for the prediction of in-hospital mortality was + 10% (sensitivity of 85.7% and specificity of 81.6%). The prediction models including CRP or P/F ratio, in addition to changes in KL-6 over 1 week, did not improve the performance of the models, including changes in KL-6 alone (Table 5). However, applying the two variables consecutively (from baseline variables to change in KL-6) was more useful for predicting in-hospital mortality (Supplementary Figure S2).

**Table 5:** Comparison of the performance of the risk prediction models for in-hospital mortality of patients with AE-ILD.

Variables	Area under curve	95% CI	*P* value	*P* value*
Baseline P/F ratio	0.713	0.550–0.876	0.005	
Baseline CRP (mg/dL)	0.818	0.728–0.909	< 0.001	
Δ KL-6, 1 week, %	0.902	0.813–0.991	< 0.001	Reference
Δ KL-6, 1 week, % + Baseline P/F ratio	0.915	0.805–0.975		0.619
Δ KL-6, 1 week, % + Baseline CRP	0.912	0.800–0.972		0.598
Δ KL-6, 1 week, % + Baseline P/F ratio + Baseline CRP	0.914	0.802–0.974		0.773

*P value compared with the risk prediction model of Δ KL-6 (1 week, %).

*AE-ILD *acute exacerbation of interstitial lung disease, *Δ* changes from baseline, *KL-6* Krebs von den Lungen-6, *P/F ratio* ratio of partial pressure of oxygen to the fraction of inspiratory oxygen, *CRP* C-reactive protein.

### Survival according to KL-6 levels

**Figure 3:**
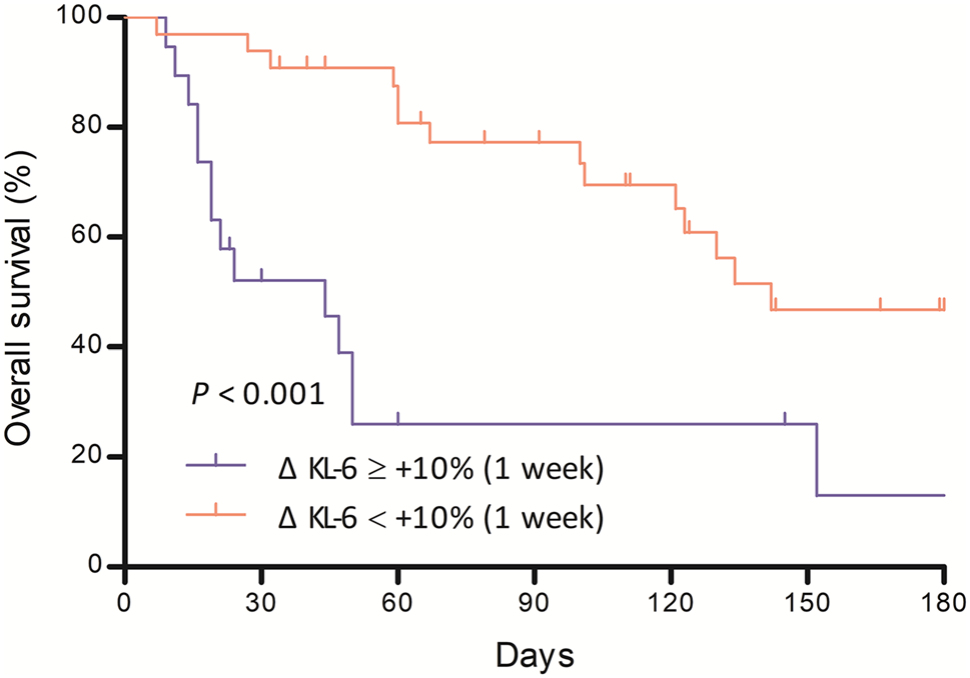
Comparison of survival curves after hospitalization between groups with high and low changes in KL-6 among patients with AE-ILD. Kaplan–Meier curves: blue line, high KL-6 change group; red line, low KL-6 change group. *KL-6* Krebs von den Lungen-6, *AE-ILD* acute exacerbation of interstitial lung disease.

When all patients were divided into two groups according to the best cut-off level for the change in KL-6 over 1 week, patients with remarkable increase in KL-6 (more than 10% relative increase from baseline) showed significantly worse survival (in-hospital mortality: 63.2 vs. 6.1%; median survival: 42 vs. 142 days; *P* < 0.001) than those without (Fig. 3).

## Discussion

To our knowledge, this is the first study to demonstrate the association between changes in blood biomarkers and the mortality of patients with AE-ILD during hospitalization. In addition to baseline CRP levels and P/F ratio, the change in KL-6 was identified as an independent prognostic factor for in-hospital mortality in ILD patients with AE. During hospitalization, a greater than 10% relative increase in KL-6 levels over 1 week could well differentiate poor prognosis among patients with AE-ILD.

In our study, a 10% relative change in KL-6 over 1 week was associated with poor prognosis of patients with AE-ILD. Previous studies also suggested the usefulness of the change in KL-6 for predicting prognosis of patients with ILD^15,24^. For 85 patients with ILD (IPF = 27, connective tissue disease [CTD]-ILD = 33, idiopathic NSIP = 18, and other idiopathic interstitial pneumonia = 7), Jiang et al. reported that a greater than 500 or 1000 U/mL increase in KL-6 within 1–6 months predicted disease progression (defined as death or decline in FVC > 10% or DLco ≥ 15% at 12 months) (odds ratio [OR] 1.73, 95% confidence interval [CI] 1.48–1.98, *P* < 0.01 and OR 2.57, 95% CI 2.26–2.88, *P* < 0.01, respectively) in the unadjusted logistic regression analysis^15^. Among 14 patients with rapidly progressive IPF (defined as deterioration of dyspnea within 2 months and the presence of ground glass opacity on HRCT), Yokoyama et al. found that, after corticosteroid pulse therapy, baseline KL-6 levels were significantly decreased at 1 or 3 weeks in survivors (− 18.9 ± 14.4%, *P* < 0.05 and − 32.7 ± 20.9%, *P* < 0.05, respectively), whereas KL-6 levels tended to increase at 3 weeks in non-survivors (+ 93.7 ± 103%; *P* < 0.05)^24^. These results indicate that a change in KL-6 level might be useful for predicting prognosis or treatment response. Moreover, unlike other biomarkers such as CRP and P/F ratio, KL-6 may more directly reflect the pathophysiology of AE-ILD characterized by diffuse alveolar damage^12,13,25^. For this reason, the change of KL-6 early in hospitalization could help to predict the prognosis of AE-ILD complementarily in addition to other biomarkers.

The baseline value of KL-6 was not associated with the prognosis of patients with IPF in our study. Contrary to our results, those of a previous study revealed the association between baseline KL-6 levels and prognosis of patients with AE-ILD^8^. For 58 patients with AE-IPF, Kishaba et al. demonstrated that high KL-6 levels at hospitalization were associated with increased risk of 90-day mortality (hazard ratio [HR] 2.909, *P* = 0.038) in the multivariable Cox analysis^8^. This contradicting result might be explained by the different clinical characteristics of the study population; our cohort had a higher P/F ratio (289.9 vs. 100–200) at hospitalization and more frequently used antifibrotic agents before AE (62.5% vs. 0%) than the cohort of the previous study^8^. Moreover, our cohort had a relatively lower mortality rate (in-hospital mortality: 24 vs. 56.9%; 3-month mortality rate: 36.1 vs. 70.7%) than the cohort of the previous report^8^. Antifibrotic agent can significantly reduce mortality after hospitalization in patients with IPF^26^; however, the previous study was not performed in the era of antifibrotic therapy^8^. Therefore, prolonged survival times in our study may reduce the predictive value of baseline KL-6 levels.

In our study, low baseline P/F ratio and high CRP levels were associated with poor prognosis of patients with AE-ILD. Previous studies have also reported similar results^8,27^. Through a multivariable Cox analysis, Kishaba et al. showed that a P/F ratio < 100 was associated with an increased risk of 3-month mortality (HR 2.42, *P* = 0.041) in patients with AE-IPF (n = 58)^8^. Cao et al. also revealed, through multivariable Cox analysis, that a lower P/F ratio at the time of hospitalization was associated with an increased risk of mortality (HR 0.989, 95% CI 0.984–0.994, *P* < 0.001) in patients with CTD-ILD (n = 70)^27^. Through unadjusted Cox analysis, Kamiya et al. reported that CRP levels at the onset of AE were associated with all-cause mortality (HR 1.05, 95% CI 1.02–1.08, *P* = 0.003) in 243 patients with AE-IPF^28^. Through multivariable logistic analysis, Song et al. also showed that CRP levels were associated with an increased risk of in-hospital mortality (OR 2.467, 95% CI 1.030–5.911, *P* = 0.043) and reduced P/F ratio (OR 0.989, 95% CI 0.983–0.996, *P* = 0.001) in patients with AE of IPF (n = 96)^4^. These results support our findings. However, unlike KL-6, the changes in CRP and P/F ratio could not predict prognosis. Thus, KL-6 was the only marker that could predict the prognosis of AE-ILD based on changes in its level over 1 week.

This study had some limitations. First, this study was of a retrospective nature and was conducted on Asian population, which may question the generalizability of our findings. Nonetheless, the baseline characteristics of patients in our study were found to be similar to those of patients in previous studies^4,15,24^. Second, only two time points (baseline and 1 week after hospitalization) were selected to evaluate the predictive role of the change in blood biomarkers. It was difficult to establish more time points during the follow-up owing to the high mortality rate of AE-ILD and the retrospective nature of this study. Nevertheless, the change in KL-6 over 1 week could well discriminate patients with poor prognosis. Third, patients with various ILDs, including IPF, were included in this study, resulting in a heterogenous distribution of diseases. However, the prognosis of AE of IPF and non-IPF ILD did not differ in a previous study^7^.

In conclusion, the relative change in KL-6 over 1 week was useful for predicting in-hospital mortality in patients with AE-ILD, in addition to baseline CRP and P/F ratio. Our data suggest that during hospitalization, a remarkable change in KL-6 level is useful for differentiating patients with poor prognosis among patients with AE-ILD.

## Supplementary Information

Supplementary Information.

## Data Availability

The datasets used and/or analyzed during the current study are available from the corresponding author upon reasonable request.
