# Supplementary material for: Changes in blood Krebs von den Lungen-6 predict the mortality of patients with acute exacerbation of interstitial lung disease
Source: Sci Rep. 2022 Mar 22;12:4916. doi: 10.1038/s41598-022-08965-9 (PMC8941048; doi:10.1038/s41598-022-08965-9)

**SUPPLEMENTARY INFORMATION**

**Changes in blood Krebs von den Lungen-6 predict the mortality of patients with acute exacerbation of interstitial lung disease**

Myeong Geun Choi^1^, Sun Mi Choi^2^, Jae Ha Lee^3^, Jung-Ki Yoon^2^, Jin Woo Song^1*^

^1^ Department of Pulmonary and Critical Care Medicine, Asan Medical Center, University of Ulsan College of Medicine, Seoul, South Korea

^2^ Division of Pulmonary and Critical Care Medicine, Department of Internal Medicine, Seoul National University Hospital, Seoul National University College of Medicine, Seoul, South Korea

^3^ Division of Pulmonary and Critical Care Medicine, Department of Internal Medicine, Inje University Haeundae Paik Hospital, Inje University College of Medicine, Busan, South Korea

Supplementary Table S1. Comparison of baseline characteristics between patients with AE-ILD according to participating centers

|  | Total | AMC | SNUH | IUHPH | *P*-value |
| --- | --- | --- | --- | --- | --- |
| Number of patients | 96 | 58 | 29 | 9 |  |
| Age, year | 70.1 ± 9.4 | 69.1 ± 8.9 | 70.1 ± 9.9 | 76.3 ± 9.1 | 0.096 |
| Male | 64 (66.7) | 40 (69.0) | 17 (58.6) | 7 (77.8) | 0.485 |
| Ever-smoker | 60 (62.5) | 41 (70.7) | 14 (48.3) | 5 (55.6) | 0.150 |
| BMI, Kg/m^2^ | 23.4 ± 3.6 | 22.5 ± 3.4 | 25.6 ± 3.5 | 22.6 ± 3.5 | <0.001 |
| Diagnosis |  |  |  |  | 0.277 |
| IPF | 58 (60.4) | 31 (53.4) | 18 (62.1) | 9 (100) |  |
| CTD-ILD | 17 (17.7) | 12 (20.7) | 5 (17.2) | 0 (0) |  |
| HP | 4 (4.2) | 2 (3.4) | 2 (6.9) | 0 (0) |  |
| Unclassified | 17 (17.7) | 13 (22.4) | 4 (13.8) | 0 (0) |  |
| Pulmonary function test |  |  |  |  |  |
| FVC, % predicted | 53.9 ± 16.5 | 51.2 ± 15.2 | 61.0 ± 21.2 | 67.8 ± 14.0 | 0.080 |
| TLC, % predicted | 58.6 ± 14.1 | 57.5 ± 12.2 | 104.0 | N/A | N/A |
| DLco, % predicted | 37.4 ± 16.9 | 34.7 ± 15.9 | 51.4 ± 22.2 | 45.2 ± 11.8 | 0.052 |
| Blood biomarkers |  |  |  |  |  |
| KL-6, U/mL | 1732.4 ± 1112.7 | 1631.4 ± 1058.2 | 2092.5 ± 1244.8 | 1190.0 ± 620.9 | 0.065 |
| LDH, IU/L | 402.6 ± 165.8 | 391.0 ± 162.3 | N/A | 476.0 ± 178.7 | N/A |
| CRP, mg/dL | 6.1 ± 7.7 | 5.9 ± 6.9 | 6.0 ± 9.2 | 8.2 ± 7.5 | 0.353 |
| P/F ratio | 289.9 ± 127.4 | 290.3 ± 123.0 | 257.5 ± 110.6 | 323.0 ± 173.8 | 0.540 |

Data are presented as mean ± standard deviation or number (%).

AE, acute exacerbation; ILD, interstitial lung disease; AMC, Asan Medical Center; SNUH, Seoul National University Hospital; IUHPH, Inje University Haeundae Paik Hospital; BMI, body mass index; IPF, idiopathic pulmonary fibrosis; CTD, connective tissue disease; HP, hypersensitivity pneumonitis; FVC, forced vital capacity; TLC, total lung capacity; N/A, not available; DLco, diffusing capacity of the lung for carbon monoxide; KL-6, Krebs von den Lungen-6; LDH, lactate dehydrogenase; CRP, C-reactive protein; P/F ratio, ratio of partial pressure of oxygen to the fraction of inspiratory oxygen.

Supplementary Table S2. Unadjusted logistic regression analysis for in-hospital mortality in patients with AE-IPF

| Variables | Odds ratio | 95% CI | *P*-value |
| --- | --- | --- | --- |
| Age | 1.067 | 0.991 – 1.148 | 0.085 |
| Male | 0.629 | 0.158 – 2.494 | 0.509 |
| Ever-smoker | 0.353 | 0.097 – 1.280 | 0.113 |
| FVC, % predicted | 1.011 | 0.966 – 1.059 | 0.631 |
| TLC, % predicted | 1.014 | 0.952 – 1.082 | 0.660 |
| DLco, % predicted | 0.941 | 0.868 – 1.020 | 0.139 |
| 6MWT, distance, m | 1.003 | 0.997 – 1.009 | 0.337 |
| 6MWT, SpO2, % | 1.014 | 0.892 – 1.153 | 0.828 |
| BAL, neutrophil, % | 1.027 | 0.984 – 1.072 | 0.221 |
| BAL, lymphocyte, % | 0.975 | 0.877 – 1.083 | 0.634 |
| Baseline KL-6 | 1.000 | 1.000 – 1.001 | 0.162 |
| Baseline LDH | 1.006 | 1.001 – 1.011 | 0.021 |
| Baseline CRP | 1.080 | 0.995 – 1.172 | 0.065 |
| Baseline P/F ratio | 0.993 | 0.987 – 1.000 | 0.052 |
| Δ KL-6, 1 week | 1.007 | 1.000 – 1.014 | 0.052 |
| Δ KL-6, 1 week, % | 1.085 | 1.013 – 1.162 | 0.019 |
| Δ LDH, 1 week | 1.011 | 0.995 – 1.028 | 0.182 |
| Δ LDH, 1 week, % | 1.147 | 0.867 – 1.517 | 0.337 |
| Δ CRP, 1 week | 0.910 | 0.798 – 1.038 | 0.159 |
| Δ CRP, 1 week, % | 1.009 | 0.995 – 1.023 | 0.217 |
| Δ P/F ratio, 1 week | 0.997 | 0.992 – 1.003 | 0.391 |
| Δ P/F ratio, 1 week, % | 1.007 | 0.997 – 1.017 | 0.150 |
| Use of antifibrotic agents ^*^ | 0.526 | 0.109 – 2.534 | 0.423 |
| Steroid pulse  ^#^ | 1.091 | 0.336 – 3.541 | 0.885 |
| Cytotoxic agent ^#^ | 5.125 | 0.766 – 34.311 | 0.092 |

^*^ At the time of hospitalization; ^#^ Treatment for AE during hospitalization

AE, acute exacerbation; IPF, idiopathic pulmonary fibrosis; FVC, forced vital capacity; TLC, total lung capacity; DLco, diffusing capacity of the lung for carbon monoxide; 6MWT, 6-minute walk test; BAL, bronchoalveolar lavage; KL-6, Krebs von den Lungen-6; LDH, lactate dehydrogenase; CRP, C-reactive protein; P/F ratio, ratio of partial pressure of oxygen to the fraction of inspiratory oxygen; Δ, changes from baseline.

Supplementary Table S3. Unadjusted logistic regression analysis for in-hospital mortality in patients with AE-non IPF

| Variables | Odds ratio | 95% CI | *P*-value |
| --- | --- | --- | --- |
| Age | 1.025 | 0.939 – 1.119 | 0.582 |
| Male | 2.179 | 0.439 – 10.830 | 0.341 |
| Ever-smoker | 2.179 | 0.439 – 10.830 | 0.341 |
| FVC, % predicted | 0.965 | 0.882 – 1.055 | 0.428 |
| TLC, % predicted | 0.824 | 0.619 – 1.097 | 0.185 |
| DLco, % predicted | 0.959 | 0.874 – 1.052 | 0.377 |
| 6MWT, distance, m | 0.999 | 0.989 – 1.010 | 0.924 |
| 6MWT, SpO2, % | 0.827 | 0.553 – 1.236 | 0.354 |
| BAL, neutrophil, % | 1.004 | 0.963 – 1.046 | 0.866 |
| BAL, lymphocyte, % | 0.899 | 0.730 – 1.107 | 0.315 |
| Baseline KL-6 | 0.999 | 0.998 – 1.000 | 0.078 |
| Baseline LDH | 1.002 | 0.997 – 1.006 | 0.507 |
| Baseline CRP | 1.395 | 1.094 – 1.778 | 0.007 |
| Baseline P/F ratio | 0.994 | 0.987 – 1.001 | 0.090 |
| Δ KL-6, 1 week | 1.008 | 0.999 – 0.018 | 0.066 |
| Δ KL-6, 1 week, % | 1.229 | 0.989 – 1.528 | 0.063 |
| Δ LDH, 1 week | 1.010 | 0.996 – 1.024 | 0.150 |
| Δ LDH, 1 week, % | 1.034 | 0.984 – 1.087 | 0.189 |
| Δ CRP, 1 week | 0.813 | 0.663 – 0.998 | 0.048 |
| Δ CRP, 1 week, % | 0.999 | 0.991 – 1.008 | 0.910 |
| Δ P/F ratio, 1 week | 0.996 | 0.989 – 1.003 | 0.225 |
| Δ P/F ratio, 1 week, % | 1.004 | 0.995 – 1.014 | 0.384 |
| Use of antifibrotic agents ^*^ | 0.333 | 0.036 – 3.119 | 0.336 |
| Steroid pulse ^#^ | 1.036 | 0.207 – 5.198 | 0.965 |
| Cytotoxic agent ^#^ | N/A | N/A | 0.999 |

^*^ At the time of hospitalization; ^#^ Treatment for AE during hospitalization

AE, acute exacerbation; IPF, idiopathic pulmonary fibrosis; N/A, not available; FVC, forced vital capacity; TLC, total lung capacity; DLco, diffusing capacity of the lung for carbon monoxide; 6MWT, 6-minute walk test; BAL, bronchoalveolar lavage; KL-6, Krebs von den Lungen-6; LDH, lactate dehydrogenase; CRP, C-reactive protein; P/F ratio, ratio of partial pressure of oxygen to the fraction of inspiratory oxygen; Δ, changes from baseline.

Supplementary Figure S1. Comparison of survival curves between patients with AE-IPF and AE-non IPF


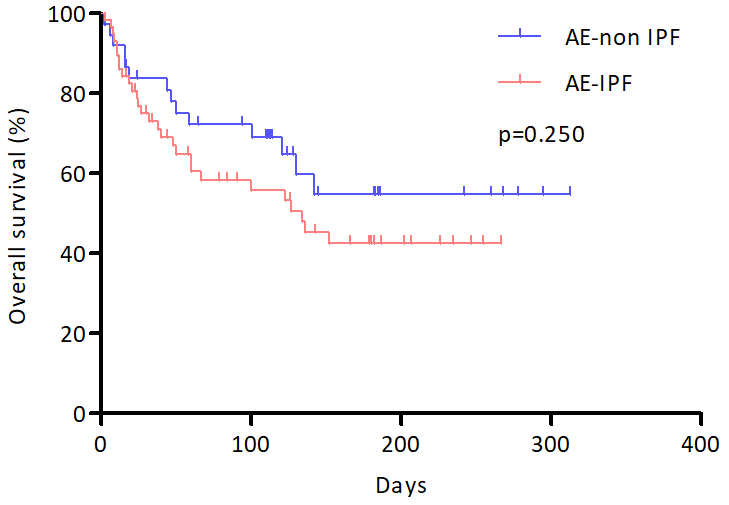


AE, acute exacerbation; IPF, idiopathic pulmonary fibrosis

Supplementary Figure S2. Sequential approach using baseline values and changes in blood biomarkers for predicting the prognosis of patients with AE-ILD.

(A) Sequential approach using baseline P/F ratio and relative change in KL-6 (%, 1 week). (B) Sequential approach using baseline CRP levels and relative change in KL-6 (%, 1 week).

The cut-off values of the P/F ratio and CRP were calculated using the best cut-off of the receiver operating characteristic curve, which best predicts the mortality of patients with AE-ILD.


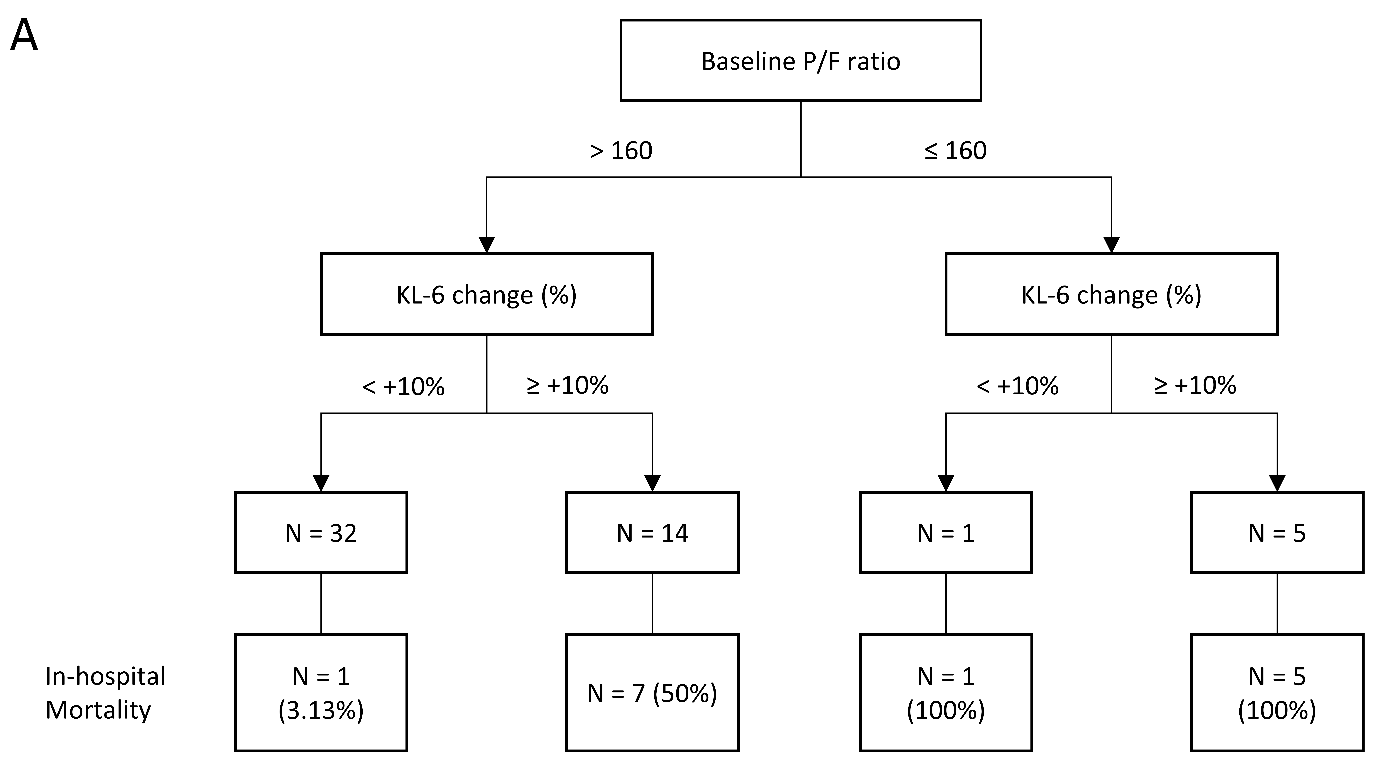
AE-ILD, acute exacerbation of interstitial lung disease; P/F ratio, ratio of partial pressure of oxygen to the fraction of inspiratory oxygen; KL-6, Krebs von den Lungen-6; CRP, C-reactive protein


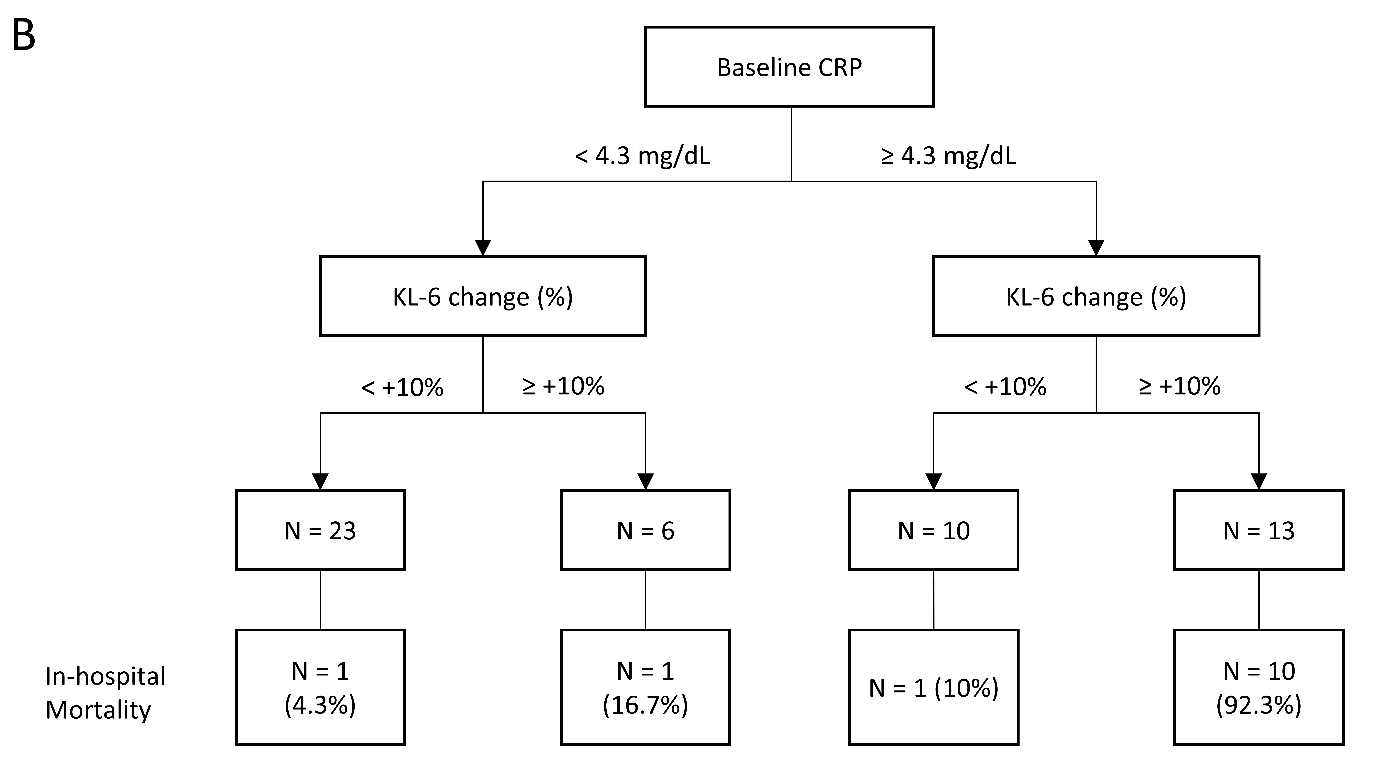

Supplement: Supplementary file 1 — Supplementary Information. [file 41598_2022_8965_MOESM1_ESM.docx]
